# Genome-wide screen for universal individual identification SNPs based on the HapMap and 1000 Genomes databases

**DOI:** 10.1038/s41598-018-23888-0

**Published:** 2018-04-03

**Authors:** Erwen Huang, Changhui Liu, Jingjing Zheng, Xiaolong Han, Weian Du, Yuanjian Huang, Chengshi Li, Xiaoguang Wang, Dayue Tong, Xueling Ou, Hongyu Sun, Zhaoshu Zeng, Chao Liu

**Affiliations:** 1 Faculty of Forensic Medicine, Zhongshan School of Medicine, Sun Yat-Sen University, Guangzhou, 510080 China; 2 Guangdong Province Translational Forensic Medicine Engineering Technology Research Center, Sun Yat-Sen University, Guangzhou, 510080 China; 3 Guangzhou Forensic Science Institute, Guangzhou, 510030 China; 4 School of Forensic Medicine, Southern Medical University, Guangzhou, 510515 China; 5 Homy Genetech Inc. Guangdong, Foshan, 52800 China; 6 Department of Forensic Medicine, School of Basic Medical Sciences, Zhengzhou University, Zhengzhou, 450001 China

## Abstract

Differences among SNP panels for individual identification in SNP-selecting and populations led to few common SNPs, compromising their universal applicability. To screen all universal SNPs, we performed a genome-wide SNP mining in multiple populations based on HapMap and 1000Genomes databases. SNPs with high minor allele frequencies (MAF) in 37 populations were selected. With MAF from ≥0.35 to ≥0.43, the number of selected SNPs decreased from 2769 to 0. A total of 117 SNPs with MAF ≥0.39 have no linkage disequilibrium with each other in every population. For 116 of the 117 SNPs, cumulative match probability (CMP) ranged from 2.01 × 10–48 to 1.93 × 10–50 and cumulative exclusion probability (CEP) ranged from 0.9999999996653 to 0.9999999999945. In 134 tested Han samples, 110 of the 117 SNPs remained within high MAF and conformed to Hardy-Weinberg equilibrium, with CMP = 4.70 × 10–47 and CEP = 0.999999999862. By analyzing the same number of autosomal SNPs as in the HID-Ion AmpliSeq Identity Panel, i.e. 90 randomized out of the 110 SNPs, our panel yielded preferable CMP and CEP. Taken together, the 110-SNPs panel is advantageous for forensic test, and this study provided plenty of highly informative SNPs for compiling final universal panels.

## Introduction

It has been well recognized that single nucleotide polymorphisms (SNPs) are potentially valuable for DNA profiling in forensics. The advantages of SNP characteristics serving as forensic markers include: (1) much higher density interspersing in the whole genome^1,2^, providing more selectable loci to offset the defect of only two alleles at each locus. (2) Shorter length of amplified fragment, facilitating the amplification of degraded DNA samples^3^. (3) Lower mutation rate, endowing them with superiority in paternity and immigration testing^4,5^. (4) Faster and cheaper high-throughput approaches available for genotyping^6–8^.

Over the past decade, studies on population genetics and forensic application of SNPs were performed. For example, Vallone *et al*.^9^ investigated the allele frequencies for 70 autosomal SNPs in U.S. Caucasian, African-American, and Hispanic populations. Kidd Lab studied hundreds of SNPs in more than 40 populations^10,11^. Moreover, several applicable panels were developed in recent years^12–15^. In retrospect, these studies selected different candidate SNPs or populations, resulting in few common loci across these panels. For instance, there are just 4 shared SNPs in the SNP*for*ID 52 and IISNP panels^12,13^, which are the best known and serve as the basis for several commercial kits, e.g. the HID-Ion AmpliSeq Identity Panel (Thermo Fisher Scientific)^16^ and the ForenSeq DNA Signature Prep Kit (https://support.illumina.com/downloads/forenseq-dna-signature-prep-guide-15049528.html). Among different ethnic populations, the vast majority of SNPs in human genome differs in minor allele frequency (MAF) and linkage disequilibrium (LD) properties, compromising to some extent the universal applicability of the panels.

To screen such common SNPs as many as possible, in this study we performed a genome-wide screen through 25,580,678 SNPs based on the databases of HapMap r28 (released in Aug 2010) and 1000 Genomes Phase 3 (released in May 2015). We collected all of the SNPs with different threshold values of MAF, and evaluated those with MAF ≥0.39 for their forensic genetic parameters. We also experimentally analyzed 117 independent SNPs with MAF ≥0.39 in two other populations — Chinese Han in Guangzhou (CHG, N = 96) and Chinese Han in Zhengzhou (CHZ, N = 38).

## Results

### Selection of universal highly informative SNPs

Based on the HapMap bulk data r28, we screened 4,402,208,440 genotypes at 25,580,678 SNPs in the whole genome. At the criterion of MAF ≥0.35, the number of SNPs shared by the 11 populations was 13466. When the threshold of MAF was set at ≥0.38–0.44, the number of shared SNPs ranged from 3881 to 21. When MAF was elevated up to 0.45, no shared SNP was retained (Table 1). After further screening through the 1000 Genomes populations (26 totally), the number of shared SNPs in the 37 populations was: 2769, 433, 169, 62, 12, 1, at the criterion of MAF ≥0.35, 0.38, 0.39, 0.40, 0.41 or 0.42, respectively; and no SNP was retained when the MAF was raised to 0.43. (Tables 2, S1). A few SNPs (rs3125289, rs311870 and rs7176637) are triallelic and the MAF is lower than 0.38, but both of the high-frequency alleles have a frequency not lower than 0.38. Such SNPs were also included in the panel of MAF ≥0.38 (Table S1). It caught our attention that in the panel of MAF ≥0.35, only one SNP (rs891700) was shared by the SNP*for*ID52^12^, and only seven (rs13218440, rs1872575, rs1554472, rs1498553, rs891700, rs1019029 and rs2291395) were shared by the IISNP panel composed of 92 SNPs developed in the Kidd Lab^13,17^ (Table S1).

**Table 1:** The number of shared SNPs by the 11 HapMap populations with different MAF.

MAF (≥)	Numbers
0.35	13466
0.38	3881
0.39	2351
0.40	1251
0.41	590
0.42	242
0.43	89
0.44	21
0.45	0

**Table 2:** The number of shared SNPs by the 37 HapMap + 1000 Genomes populations with different MAF.

Chromosome	MAF (≥)						
	0.35	0.38	0.39	0.40	0.41	0.42	0.43
1	171	23	11	2	1	0	0
2	199	21	10	5	0	0	0
3	235	39	10	2	0	0	0
4	176	38	13	5	0	0	0
5	280	30	12	4	1	0	0
6	195	33	23	11	1	1	0
7	161	34	12	4	0	0	0
8	161	27	9	2	0	0	0
9	136	14	4	2	0	0	0
10	100	6	2	0	0	0	0
11	125	18	9	2	0	0	0
12	135	25	3	0	0	0	0
13	97	20	12	2	0	0	0
14	86	11	2	0	0	0	0
15	67	8	2	0	0	0	0
16	54	11	6	4	2	0	0
17	101	14	4	1	0	0	0
18	76	18	6	3	0	0	0
19	34	4	0	0	0	0	0
20	69	15	9	7	3	0	0
21	67	16	9	6	4	0	0
22	26	4	0	0	0	0	0
X	18	4	1	0	0	0	0
Y	0	0	0	0	0	0	0
Sum	2769	433	169	62	12	1	0

### Genetic investigation of the SNPs with MAF ≥0.39

We studied the population genetic profiles of the SNPs in the panel of MAF ≥0.39, which includes 169 SNPs. Significant deviation of genotype frequency from expectations was observed in 366 out of 6253 HWE tests in the 37 populations (*P* < 0.05, Table S2). After the Bonferroni’s correction, the deviation remained in 34 tests (*P* < 0.0003, Table S2). There was no LD between at least 117 out of the 169 SNPs in each of the populations (r^2^ < 0.05, Table S3). We selected 117 independent SNPs (including one X-linked SNP rs722847, and only one of those with r^2^ ≥ 0.05 between each other in any of the populations was selected) to evaluate forensic parameters (Table S3). For the 116 autosomal SNPs, MP ranged from 0.333 (rs7127767 in HapMap-MEX) to 0.529 (rs7561460 in 1000 Genomes-PEL), and CMP ranged from 2.01 × 10^−48^ in HapMap-ASW to 1.93 × 10^−50^ in 1000 Genomes-STU (Table S4). EP ranged from 0.012 (rs2624459 in HapMap-JPT) to 0.419 (rs7561460 in 1000 Genomes-PEL), and CEP ranged from 0.9999999996653 in 1000 Genomes-STU to 0.9999999999945 in 1000 Genomes-ASW (Table S4). For the X-linked SNP rs722847, calculated from the genotypes in females, MP ranged from 0.336 in HapMap-MKK to 0.445 in 1000 Genomes-CHS, and EP was between 0.030 in HapMap-CHB and 0.295 in 1000 Genomes-CHS (Table S4).

### Experimental studies on population genetics of the 117 SNPs with a MAF ≥0.39

We further investigated the 117 SNPs in two Chinese Han groups including CHG and CHZ. In the groups, 112 out of the 117 SNPs were successfully genotyped among all the samples (genotyping rate: 100%), 3 SNPs (rs508485, rs530913, and rs10451160) were with a genotyping rate of 99.3%, and 2 SNPs (rs6136874 and rs10503926) were 97.8%. A total of 89 SNPs remained a MAF of ≥0.39 in both groups (Table S5), as did 109 when the two groups were pooled (CHP, Table S5). Four SNPs (rs6431272, rs431951, rs4469483, rs4487849) exhibited significant deviation of genotype frequency from HWE expectations after Bonferroni’s correction in CHG, CHZ and CHP (P < 0.000428, Table S6), as did the other two SNPs (rs10451160 and rs7710223) in CHG and CHP. The observed range of heterozygosities was 0.39583–0.68750, 0.34211–0.65789, 0.41791–0.65672, respectively in CHG, CHZ and CHP, except for the SNPs deviating significantly from HWE expectations (Table S6). Out of 40716 pairwise comparisons, 2040 pairs showed significant LD (data not shown). All of the LDs were most likely due to chance, because the paired SNPs locate on different chromosomes, or on the same chromosomes but there are other SNPs showing no LD between them.

**Figure 1:**
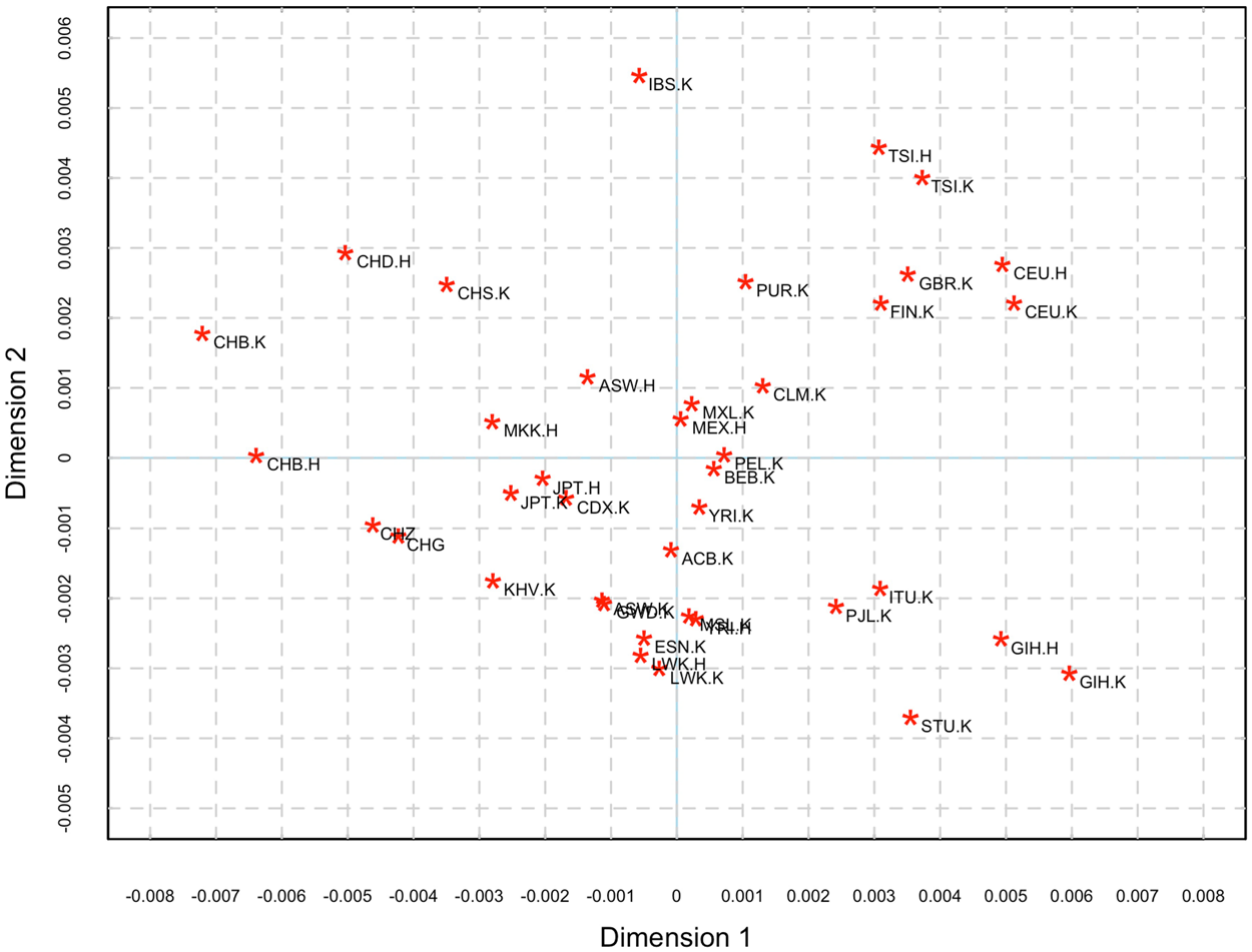
Multidimensional scaling plot drawn from genetic distance calculated between 39 worldwide populations analyzed for 110 autosomal SNPs. H or K after a dot represents the populations from the HapMap or 1000 Genomes databases respectively.

Results of AMOVA analysis showed that the global genetic variation in CHG and CHZ could be explained by individual variability (Table S7), suggesting the reasonability of combining CHG and CHZ. Moreover, the analysis of genetic distance for the 110 autosomal SNPs without significant deviation from HWE revealed tiny variations between all of the studied populations (Table S8, Fig. 1), proving the universality of these SNPs.

We further selected the 110 autosomal SNPs for calculating forensic parameters. MP ranged from 0.334 (rs12819145 and rs1293288 in CHZ) to 0.494 (rs2624459 in CHZ) (Table S9), and CMP ranged from 4.70 × 10^−47^ in CHP to 8.37 × 10^−46^ in CHZ (Tables 3 and S9). EP ranged from 0.082 (rs2895309, rs12819145, rs1293288 and rs2380437 in CHZ) to 0.366 (rs2624459 and rs4607417 in CHZ) (Table S9), and CEP ranged from 0.999999999862 in CHP to 0.999999999939 in CHZ (Tables 3 and S9). The commercialized HID-Ion AmpliSeq Identity Panel (currently named Precision ID Identity Panel) includes 90 autosomal SNPs and was recently evaluated in Chinese Han population^16^. CMP of this panel and the PowerPlex Fusion STR System were 1.318 × 10^−33^ and 2.147 × 10^−25^, respectively; and CEP were 0.999999724 and 0.999999999673, respectively (Table 4)^16^. Here, in CHP, CMP calculated for 90 randomized out of the 110 SNPs with a MAF of ≥0.39 ranged from 3.071 × 10^−39^ to 5.368 × 10^−38^, at least 10^−5^ lower than that achieved with these two commercial panels; CEP ranged from 0.9999999728 to 0.99999999736, higher than that of HID-Ion AmpliSeq Identity Panel and lower than that of PowerPlex Fusion STR System (Table 4). These results suggest that the MAF ≥0.39 panel would have obvious advantages in forensic application over HID-Ion AmpliSeq Identity Panel. When the 110 SNPs are all included, it would have overall advantage than PowerPlex Fusion STR System.

**Table 3:** Forensic parameters calculated for 110 SNPs in CHG, CHZ and CHP.

Population	CHG	CHZ	CHP
CMP	9.220 × 10^−47^	8.366 × 10^−46^	4.697 × 10^−47^
CEP	0.999999999896	0.999999999939	0.999999999862

CHG, Chinese Han in Guangzhou; CHZ, Chinese Han in Zhengzhou; CHP, the pooled population with CHG and CHZ.

**Table 4:** Forensic parameters calculated for two commercialized panels and 90 randomized SNPs included in the MAF ≥0.39 panel in Chinese Han population.

Panel	MAF ≥0.39 panel	HID-Ion AmpliSeq Identity Panel^16^	PowerPlex Fusion STR System^16^
Number of autosomal loci	90	90	22
Number of individuals	134	108	108
CMP	3.071 × 10^−39^–5.368 × 10^−38^	1.318 × 10^−33^	2.147 × 10^−25^
CEP	0.9999999728–0.99999999736	0.999999724	0.999999999673

## Discussion

An expanded genome-wide search in this study contributes to the identification of more and probably better forensic markers. Our results indicated that high-throughput SNPs databases can provide convenient, efficient and cost-saving approaches to select highly informative SNPs for forensic purposes. With these approaches, this study obtained several panels with such candidate SNPs, which have potential to be used to develop final forensic SNP panels with universal applicability in a variety of ethnic populations. Until now, three individual identification SNP kits are commercially available, including the HID-Ion AmpliSeq Identity Panel^16,18^, the ForenSeq DNA Signature Prep Kit^19^ and the Qiagen SNP-ID Kit^20^. They are all mainly based on the SNP *for* ID52^12^ and Kidd IISNP panels^13,17^, which have no SNP in common with the MAF ≥0.39 panel, and just 8 with the MAF ≥0.35 panel (Tables S1 and S9). Comparison shown in Table 4 indicated that the MAF ≥0.39 panel is potentially advantageous in forensic application.

A most recent study by Li *et al*.^21^ also performed a genome-wide screening by selecting SNPs with Fst ≤0.01 and heterozygosity ≥0.40, based on HapMap r24, which includes 4 populations. It proposed a final panel encompassing 175 SNPs, compiled based on 26 populations (four in HapMap r24, thirteen in 1000 Genomes phase 1, nine Chinese populations including five Han). A certain MAF/heterozygosity value was not set as a screening criterion after the initial screening in HapMap r24. As a result, MAF values of these 175 SNPs differed greatly: in the range of 0.08–0.50 in 1000 Genomes phase 3, 0.20–0.50 in the nine Chinese populations, and allele frequencies for 69 out of the 175 SNPs were not available in HapMap r28. In our study, MAF ≥0.35, 0.38, 0.39, 0.40, 0.41, 0.42….. was set as a screening criterion in all of the HapMap r28 (includes all of the 4 populations in r24) and 1000 Genomes phase 3 populations. The selected loci were all high polymorphic in all of the studied populations. Consequently, the final panel in Li’s study^21^ includes just 7 SNPs in common with our MAF ≥0.35 panel.

In conclusion, our study provided several semi-finished panels, which are convenient for researchers to select candidate high polymorphic SNPs to further test in more ethnic lines for the purpose of compiling final universal panels.

## Methods

### Genome-wide screening for highly informative SNPs

Bulk data of the whole genome-genotyped SNP allele frequencies of the HapMap Public Release #28 were downloaded from the website http://hapmap.ncbi.nlm.nih.gov/downloads/frequencies/2010-08_phaseII+III/. These data include genotyping results of the whole genome-wide SNPs in 1301 individuals from 11 ethnical populations. A method described in our previous studies was used to select candidate SNPs^22,23^. Briefly, frequency files in.gz format were unzipped and read by Microsoft Excel 2007 (Microsoft Corp., Redmond, WA). Rs numbers corresponding to SNPs at certain MAF criterion (i.e., MAF ≥0.45, 0.44, 0.43, 0.42, 0.41, 0.40, 0.39, 0.38, 0.35) were listed in a new.xlsx file containing one column for each population. Rs numbers shared by all 11 columns were screened out by a program (for program codes, please refer to Supplementary materials in Table S10).

All of the selected SNPs were further screened with the same MAF value in the database of 1000 Genomes Phase 3 at http://browser.1000genomes.org/index.html. The database includes genotyping results of the whole genome-wide SNPs in 2504 individuals from 26 ethnical populations. All of the populations studied were listed in Table S10. SNPs shared by all of the 26 populations at a MAF criterion were selected.

### Ethnics

Blood samples were collected from the CHZ and CHG populations upon approval of the Ethics Committee at Zhongshan School of Medicine, Sun Yat-Sen University. Informed consent was obtained from all participants. All the experimental procedures were carried out in accordance with the approved guidelines of Zhongshan School of Medicine, SunYat-Sen University. This study was approved by the Ethics Committee of Zhongshan School of Medicine, SunYat-Sen University.

### Genotyping assay

Genotypes of the 117 SNPs without LD with each other in the panel of MAF ≥0.39 were analyzed using the MassARRAY Genetic Analysis System (Sequenom, San Diego, California, USA). Biochemical reactions were performed in four wells. Primers were designed using the software AssayDesigner version 3.1. Reaction reagents and program were used as we previously described^24^. Briefly, the polymerase chain reaction (PCR) volume was composed of 0.5 μL of 10 × PCR buffer, 0.4 μL of 25 mM MgCl_2_, 1 μL of 0.5 μM primer Mix, 0.1 μL of 25 mM dNTP Mix, 1 μL of 10 ng/μL DNA template, 0.2 μL of 5 U/μL HotStar Taq DNA polymerase and 1.8 μL of water. The SAP reaction volume contained 0.17 μL of SAP buffer, 0.3 μL of 1.7 U/μL SAP enzyme, 1.53 μL of nanopure water. The extension reaction reagents included 0.2 μL of iPLEX Buffer Plus, 0.2 μL of iPLEX Termination Mix, 0.94 μL of iPLEX Extend Primer Mix, 0.041 μL of iPLEX Enzyme, 0.619 μL of nanopure water, 7 μL of PCR + SAP product. The extension reaction program was set as: initial denaturing in 94 °C for 30 s, then denaturing in 94 °C for 5 s, five cycles (we termed mini-cycle) of annealing in 52 °C for 5 s and extension in 80 °C for 5 s. Totally 40 cycles of the denaturing and mini-cycle, followed by final extension in 72 °C for 3 min. Twenty samples were randomly selected for Sanger sequencing to verify the genotyping results. All of the sequencing results were consistent with that of the MassARRAY method.

### Statistical analyses

Results of LD analysis of SNPs in the 1000 Genomes populations were online searched at http://www.ensembl.org/Homo_sapiens/Variation. Deviations of genotype frequencies from Hardy-Weinberg equilibrium (HWE) expectations for intra-population were tested using the modified PowerStats (Promega, Madison, WI, USA). Forensic parameters such as random match probability (MP), cumulative random match probability (CMP), exclusion probability (EP), cumulative exclusion probability (CEP) were estimated also using the modified PowerStats. Similar statistical analyses for experimental populations were performed using the Arlequin version 3.5^25^. A two-hierarchical AMOVA analysis was performed to study the degree of genetic heterogeneity between the experimental populations. A multidimensional scaling analysis was performed from genetic distance using the SPSS version 22.0.

## Electronic supplementary material

Dataset 1

Dataset 2

Dataset 3

Dataset 4

Dataset 5

Dataset 6

Dataset 7

Dataset 8

Dataset 9

Dataset 10
