# Supplementary material for: Genome-wide screen for universal individual identification SNPs based on the HapMap and 1000 Genomes databases
Source: Sci Rep. 2018 Apr 3;8:5553. doi: 10.1038/s41598-018-23888-0 (PMC5882920; doi:10.1038/s41598-018-23888-0)
Supplement: Supplementary file 7 — Dataset 7 [file 41598_2018_23888_MOESM7_ESM.doc]

**Result of AMOVA analysis performed for 117 SNPs in CHG and CHZ**

| Source of variation | d.f. | Sum of squares | Variance components | Percentage of variation |
| --- | --- | --- | --- | --- |
| Between populations | 1 | 40.962 | 0.10638 Va | 0.37 |
| Among individuals within populations | 132 | 3877.915 | 0.66109 Vb | 2.29 |
| Within individuals | 134 | 3759.500 | 28.05597 Vc | 97.34 |
| Total | 267 | 7678.377 | 28.82343 |  |
| Fixation Indices | | | | |
| FIS: 0.02302 | | | | |
| FST: 0.00369 | | | | |
| FIT: 0.02663 | | | | |

Significance tests (1023 permutations)

Vb and FIS : P(rand. value > obs. value) = 0.17204

P(rand. value = obs. value) = 0.00098

P-value = 0.17302+-0.01072

Va and FST : P(rand. value > obs. value) = 0.78788

P(rand. value = obs. value) = 0.00000

P-value = 0.78788+-0.01192

Vc and FIT : P(rand. value < obs. value) = 0.12317

P(rand. value = obs. value) = 0.00098

P-value = 0.12414+-0.00987

Population specific FIS indices (1023 permutations)

-----------------------------------------------------------------------------

Pop# Name FIS P(Rand FIS>=Obs FIS)

-----------------------------------------------------------------------------

1 G001 0.02093 0.218964

2 Z001 0.02834 0.247312

-----------------------------------------------------------------------------
